# Interfacial Engineering of SeO Ligands on Tellurium Featuring Synergistic Functionalities of Bond Activation and Chemical States Buffering toward Electrocatalytic Conversion of Nitrogen to Ammonia

**DOI:** 10.1002/advs.201901627

**Published:** 2019-08-20

**Authors:** Gong Zhang, Hang Xu, Yang Li, Chao Xiang, Qinghua Ji, Huijuan Liu, Jiuhui Qu, Jinghong Li

**Affiliations:** ^1^ School of Environment State Key Joint Laboratory of Environment Simulation and Pollution Control Tsinghua University Beijing 100084 China; ^2^ Center for Water and Ecology Tsinghua University Beijing 100084 China; ^3^ Key Laboratory of Integrated Regulation and Resource Development on Shallow Lake of Ministry of Education College of Environment Hohai University Nanjing 210098 China; ^4^ Department of Chemistry Key Laboratory of Bioorganic Phosphorus Chemistry & Chemical Biology Tsinghua University Beijing 100084 China

**Keywords:** bond activation, charge reservoir, electrocatalytic nitrogen reduction reaction (NRR), incorporation, tellurium

## Abstract

Ammonia (NH_3_) production from electrochemical nitrogen (N_2_) reduction reaction (NRR) under ambient conditions represents a sustainable alternative to the traditional Haber–Bosch process. However, the conventional electrocatalytic NRR process often suffers from low selectivity (competition with the hydrogen evolution reaction (HER)) and electron transfer bottleneck for efficient activation and dissociation. Herein, a strategy to simultaneously promote selectivity and activity through dual‐incorporation of Se and O elements onto the shell of HER‐inactive Te nanorods is reported. It is theoretically and experimentally verified that the exposure of lone‐pair electrons in the TeO_2_ shell of Se, O dual‐doped Te nanorods can maximize orbits overlap between N_2_ and Te for N‐N bond activation via π‐backdonation interactions. Further, the Gibbs free energy change indicates that the Lewis‐basic anchor ‐SeO ligand with strong electron‐donating characteristics serves as an electron reservoir and is capable of buffering the oxidation state variation of Te, thereby improving the thermodynamics of desorption of the intermediates in the N_2_‐to‐NH_3_ conversion process. As expected, a high faradaic efficiency of 24.56% and NH_3_ yield rate of ≈21.54 µg h^−1^ mg^−1^ are obtained under a low overpotential of ≈0.30 V versus reversible hydrogen electrode in an aqueous electrolyte under ambient conditions.

## Graphical abstract

**A strategy that is capable of efficient N‐N bond activation and buffering the oxidation state variation of the active sites** is designed through a dual‐incorporation of Se, O elements onto the shell of hydrogen evolution reaction‐inactive Te. This ideal catalytic platform exhibits high faradaic efficiency of 24.56% and an NH_3_ yield rate of 21.54 µg h^−1^ mg^−1^ in aqueous electrolyte.

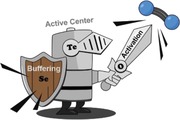

Electrocatalytic nitrogen‐to‐ammonia fixation is emerging as a sustainable strategy to tackle the energy‐intensive and high‐pressure hydrogen operations of the conventional Haber–Bosch process for ammonia production.^1^ Exploration of catalysts to increase selectivity, enhance the rate, and decrease energy consumption for desorption of key intermediates from surface should be the key to electrocatalytic N_2_ fixation.^2^ Due to the preferential adsorption of H atoms over nitrogen (N) atoms on traditional metal sites, electrochemical N_2_ fixation using the conventional metallic elements containing catalysts is incapable of achieving a satisfactory efficiency.^3^ The faradaic efficiency (FE) for aqueous reactions using the current nitrogen reduction reaction (NRR) catalysts has been usually less than 15%, and NH_3_ yield rates among these catalysts were seldom more than 20.00 µg h^−1^ mg^−1^ in previous electrochemical N_2_ reduction processes.^4^ Although several metals located at the left side of the Sabatier principle volcano plot bind N more strongly than H, the limited NRR activities were due to the fact that desorption of intermediates was too slow.^5^ Therefore, primary principle for NRR catalyst construction is high selectivity, whereby the selected element should has an unfavorable H adsorption energy and appropriate binding to nitrogen species.

Previous research has found that chemisorption of gas‐phase N_2_ onto catalysts surface is the prerequisite for subsequent N_2_ activation.^6^ The binding strength with N_2_ mainly originates from advantageous combination of occupied and empty orbitals, and the “acceptance−donation” of electrons is the nature of interaction toward N_2_.^7^ Interfaces with these specific orbitals have been deliberately designed to accept lone‐pair (LP) electrons in N_2_, whereby electrons can be donated into antibonding orbitals of N_2_ to weaken the N‐N triple bond (high dissociation energy of 941 kJ mol^−1^).^8^ For possible application, we need to modulate optimized electronic structure according to the reaction thermo‐dynamics and kinetics, whereby active sites with appropriate molecular orbitals enable N_2_ to reach an optimum activation energy for subsequent activation step.

Of the oxygen‐family elements, hydrogen evolution reaction (HER)‐inactive tellurium (Te) is a semiconductor that shows high electrical conductivity in certain directions.^9^ The electronic configuration of Te is 5s25p4 and the abundant s, p orbitals are capable of providing adequate empty or unoccupied orbitals for accepting σ‐bonding electrons in N_2_, so that N_2_ molecules are readily to be anchored through bonding interaction.^10^ Further, as shown in **Figure** **1**a, it is theoretically predicted that the incorporation of O atom into Te catalytic centers resulting in exposure of LP electrons in the p‐block element leads to a substantially elongated N‐N bond as Te centers act in a “pushpull” fashion to activate N≡N bond (Figure 1b). The general chemical motif of O = Te⟨OO⟩ Te ⟨OO …⟩Te = O chains is reflected in the TeO_2_ structure.^11^ The short Te‐O bond (≈1.9 Å) is termed equatorial bond (Te‐O_eq_), while long Te‐O bond (≈2.15 Å) is perpendicular to equatorial plane and is denoted as axial bond, Te‐O_ax_. In contrast to the covalent nature of Te‐O_eq_, the weak Te‐O_ax_ bond can be considered as an intermolecular contact. After elongating the Te‐O_ax_, a positive intermolecular overlap between lowest unoccupied molecular orbital (LUMO) of neighboring TeO_2_ unit could endow the molecular orbitals (MO) with bonding character, progressively lowering LUMO energy for better attracting the σ‐bonding electrons of N_2_ (Figure 1c),^12^ and N_2_ will be thereafter activated to the necessary intermediate [OTe]N_2_^−1^. However, O ligand is not capable of serving electron reservoir for buffering oxidation state variation of Te element, thereby restricting rapid desorption of intermediates in N_2_‐to‐NH_3_ process.

**Figure 1:**
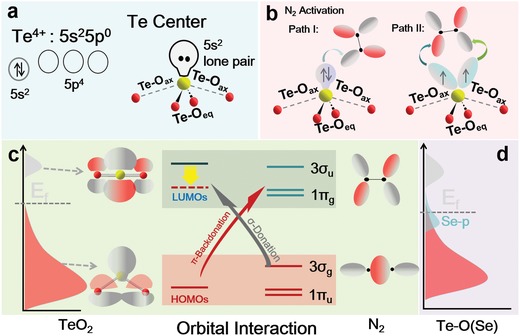
N_2_ adsorption and activation. a) Electronic configuration of Te^4+^ and TeO_2_. b) Activation of N_2_ using TeO_2_ through backbonding interactions. Path I: π‐backdonation to N_2_ molecule in an end‐on manner. Path II: π‐backdonating in side‐on manner. c) Adsorption and activation of N_2_ molecules on TeO_2_ after elongating Te‐O_ax_ bond via bonding and backbonding interactions. d) Schematic band diagrams of TeO_2_ after introduction of Se.

To surmount these obstacles, it is plausible that the incorporation of highly electron‐donating elements may realize the synergistic modulations of both adsorption and activation sites in order to functionalize N_2_ molecule.^13^ By bearing this fact in mind, we propose that the incorporation of Se into TeO_2_ shell may be an effective approach to achieve an appropriate electronic structure for better N_2_ fixation process. As shown by calculated density of states (DOS) in Figure S1 in the Supporting Information, the hybridization between Te 5p‐orbital and O 2p‐orbital is remarkably enhanced after introducing Se, leading to an increased DOS near Fermi level, which brings about more charge carriers for conductivity (Figure 1d). Theoretical calculation predicted that substitution of Se for O in Te‐O_eq_ bonds would have positive influence on the Te‐O_ax_ bond length, thereby enhancing bonding effect toward N_2_. More importantly, considering the importance of backdonation bonding between active centers and Lewis‐basic anchor in NRR process, the SeO ligands with strong electron‐donating character have the potential to act as electron reservoirs, thereby buffering oxidation state variation, so that thermodynamics of protonation/reduction process should be effectively improved in N_2_‐to‐NH_3_ conversion process.

Motivated by the promising theoretical prediction, Te nanowires (NWs) were prepared by the disproportionation of Na_2_TeO_3_ in ammonia solution at ≈160 °C. After addition of Se (Na_2_SeO_3_)‐containing diluted hydrazine hydrate, Se, O atoms were initially released from precursor and inserted into rigid Te skeleton, resulting in a series of Se, O‐dual‐element‐doped Te materials (Figure S2, Supporting Information).^14^ The morphologies of the pristine Te NWs and Se, O‐dual‐decorated Te were meanwhile characterized by electron microscopy. As shown in **Figure** **2**a, Te NWs have a highly uniform diameter of ≈10 nm and length of hundreds nanometers. High‐resolution transmission electron microscopy (TEM) images evidenced that Te NWs have a smooth surface along the length, with the axis of NWs along [001] direction (Figure S3, Supporting Information).^15^ After introduction of NaSeO_3_, wire‐structured products were converted into rods, with the diameter increased to ≈20 nm and length decreased to ≈100 nm (Figure 2b).

**Figure 2:**
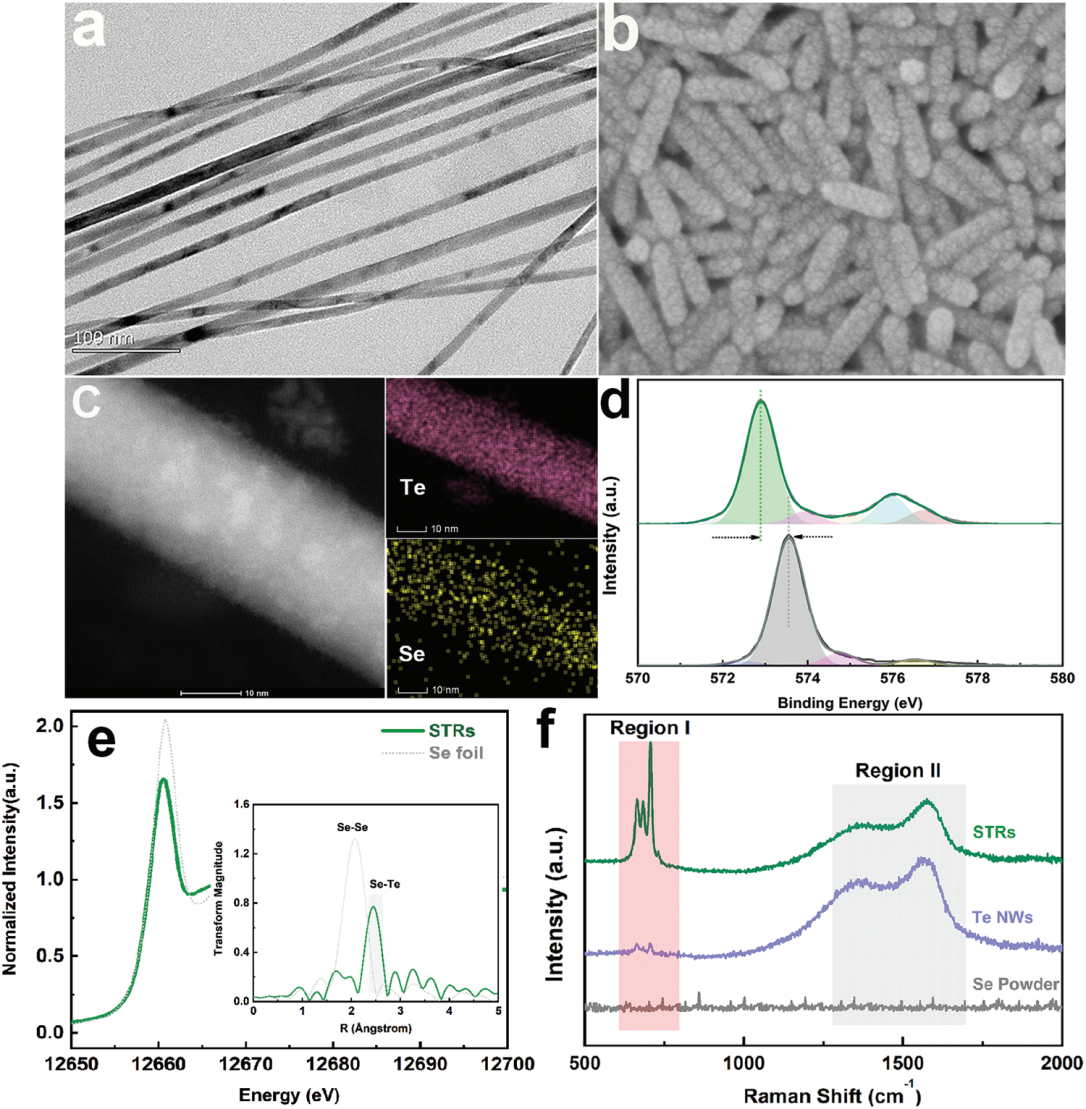
Structure and composition characterizations for Te‐based materials. a) Scanning electron microscope (SEM) images for Te NWs. b) SEM images for as‐obtained STRs. c) Elemental mapping images of Se, Te, and O on surface of STRs. d) Comparison of high‐resolution Te 3d XPS spectra for Te NWs (gray line) and STRs products (green line). e) Se K‐edge XANES and corresponding EXAFS spectra (inset) of STRs and Se reference. f) Raman spectra of STRs, Te NWs, and Se powder, respectively.

The composition of Se, O‐decorated Te nanorods (STRs) was investigated by the inductively coupled plasma‐mass spectrometry. By fixing the dosage of Te precursor, the mass fraction of Se in the STRs could be controllably tailored via increasing the amount of the Se precursors (Table S1, Supporting Information). To ascertain atomic incorporation of Se, we performed energy dispersive X‐ray spectroscopy mapping on STRs. As depicted in Figure 2c, the elements of Te, Se, and O corresponding to scan TEM (STEM) image were well distributed through the whole nanorod, manifesting a uniform elemental doping during the formation process. The characteristic peaks of X‐ray diffraction patterns for all samples could be well‐indexed to that of crystalline Te (Figure S4, Supporting Information).

The chemical structures of the different samples were confirmed from X‐ray photoelectron spectroscopy (XPS) spectra. As shown by Te 3d XPS spectra in Figure 2d, the pristine Te NWs exhibit by both an elemental Te 3d5/2 peak (≈572.80 eV) and a trace amount of oxidized tellurium (≈575.95 eV), in good agreement with previous reports.^16^ In the presence of Se and O elements, Te 3d peak in STRs showed an obvious shift, which indicated that the Se, O‐decorated Te nanostructures contained a different valence state of Te. Meanwhile, the intensity of peak at ≈575.95 eV assigned to Te^4+^ was remarkably increased with the growth of Se (Figure S5, Supporting Information), suggesting that surface coating of Se and O could alter the chemical state of Te NWs. To gain insight into the atomic configuration of the elements Te, Se, and O, comparative Raman spectra of pristine Te NWs and STRs were subsequently investigated (Figure 2f). For Te NWs, the characteristic Raman peaks in Region II were resulted from lattice vibration of Te NWs, while weakly perceptible peak in Region I was derived from the Te‐O vibration.^17^ In contrast, Raman spectra for STRs exhibited intense peaks at ≈665, 684, and 707 cm^−1^ in Region I. Strong peaks at 665 and 707 cm^−1^ might be assigned to intensified Te‐O vibration in shell layer, while the new appeared peak at 684 cm^−1^ should be involved in these Se‐based chemical bonds. In contrast to Raman spectra for pristine Se powder, the lack of characteristic Raman peaks at 236 and 458 cm^−1^ excluded the presence of chain‐structured and annular Se in the STRs (Figure S6, Supporting Information).^18^ Thus, the new peak at ≈684 cm^−1^, to a great extent, was ascribed to Te‐Se‐O vibrations.

To further verify this hypothesis, Se K‐edge X‐ray absorption spectroscopy measurements were carried out, and Figure 2e shows Se K‐edge X‐ray absorption near edge structure (XANES) spectra of STR samples as well as Se (0) powder as reference. In principle, the energy position of the absorption edge is indicative of the average valence of Se in a material.^19^ Energy position of main peak (white line) for the STRs (12 600 eV) is much lower than that of Se (IV) (12 664 eV), which is even slightly lower than Se (0) (12 601 eV) standard. This indicated that Se must be exist in low valence state in STRs. Extended X‐ray absorption fine structure spectroscopy (EXAFS) measurements at Se K‐edge were carried out to investigate the local structure. Fourier transforms for Se present a prominent peak in a range of 1.5–2.5 Å, which is corresponding to the Se–Se correlation.^20^ The peak slightly red shifts to a higher value in STRs, which was derived from Te–Se coordination. Meanwhile, O K‐edge XANES spectra of products were recorded using total‐electron‐yield mode, studying the electron excitations from O 1s orbital to unoccupied states. In comparison with pristine TeO_2_, intensity of a peak at ≈533 eV assigned to Te‐O antibonding orbits markedly decreased in STRs, which was unambiguously due to charge redistribution (accumulation of O 2p electrons) induced by the hybridization with low valence state Se (Figure S7, Supporting Information).

As is all well‐known, in addition to requirement of suitable electroconductivity, the adsorption of N_2_ onto catalyst is the first step to initialize NRR and the adsorption manner determines subsequent reduction reaction path.^21^ Therefore, the positive effect of Se, O incorporation on the anchoring of N_2_ molecular was quantitatively investigated. The weak charge redistribution after loading of N_2_ indicates that weak adsorption of N_2_ on pristine Te, ruling out the possibility of effective electrocatalytic activation. After introducing a TeO_2_ shell, the N_2_ can be favorably adsorbed on surface in an end‐on configuration via the bonding reaction with Te center, with the adsorption value decreased to ≈−0.15 eV. More importantly, the charge density distribution results confirm that the incorporation of Se is capable of intensifying the effective charge in comparison with that in Te/TeO_2_, suggesting that Se can enable more efficient charge transfer from Te to N_2_. As shown in **Figure** **3**b, remarkable charge accumulation toward N_2_ and rapid charge depletion near to Te atom verify strong electron transfer behavior between Te‐Se‐O and N_2_, which further decreased the N_2_ adsorption energy to ≈−0.18 eV.

**Figure 3:**
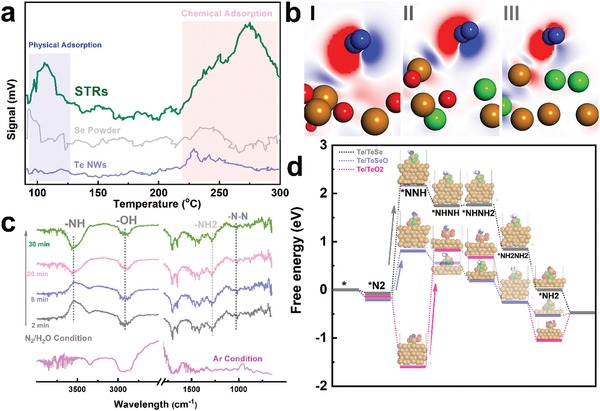
Catalytic N_2_‐to‐NH_3_ conversion analysis. a) Comparison of N_2_ adsorption on surface of different materials. Data from N_2_‐TPD profiles are shown for STRs (green), Te (blue), and Se (gray). b) Charge density difference of N_2_‐adsorbed TeO_2_ (I), TeSeO (II), and TeSeO (III). Red and blue isosurfaces, respectively, represent charge accumulation and depletion in space. c) In situ DRIFT spectra of STRs after treatment with N_2_/H_2_O at room temperature. d) Illustration of free energy diagram for Te‐containing samples. Te, O, Se, and H atoms are shown in gold, blue, and white, respectively.

Temperature‐programmed desorption (TPD) measurements were further investigated to confirm effective adsorption of N_2_ onto Se‐decorated Te/TeO_2_. N_2_ desorption isotherms in Figure 3a reveal that a peak appears at ≈100 °C that can be ascribed to desorption of physically adsorbed N_2_, whereas the signal at ≈250 °C is related to chemisorbed N_2_.^22^ In general, a hierarchical or porous‐structured catalyst should have a positive influence on the performance of N_2_ adsorption as it may facilitate N_2_ diffusion, thereby improving utilization of active sites (Figure S8, Supporting Information). In contrast to Te NWs, the STRs catalyst with a higher Brunauer–Emmett–Teller surface area provided more diffusion opportunities for attracting the molecular N_2_ via strengthened σ‐bonding interaction, which was reflected by increased physical‐adsorption peak at ≈100 °C. Furthermore, in well agreement with theoretical prediction, the other typical desorption peak of STRs catalyst can be clearly identified at 250 °C, suggesting that N_2_ molecules are bind more strongly with Te‐O sites in STRs.

To dig deeper into chemical adsorption of N_2_ onto STRs, and to gain more mechanistic insights, in situ diffuse reflectance infrared Fourier transform spectroscopy (DRIFTS) studies were thereafter carried out. The samples were treated in DRIFTS sample holder under N_2_/H_2_O or Ar atmosphere at room temperature. Figure 3c demonstrates time‐dependent changes in the in situ DRIFT spectrum after injection of N_2_. Broad band ≈3000 cm^−1^ and peak at ≈1700 cm^−1^ can be assigned to ν(O−H) stretching mode and σ(O−H) bending mode.^23^ Considering that the NH_3_ evolution via multistep proton‐coupled electron transfer process, strong signal at ≈3500 cm^−1^ should be ascribed to stretching vibrations of N−H in intermediates.^24^ In particular, the peaks appearing in range from 1250 to 1500 cm^−1^ can be attributed to H−N−H bending (≈1450 cm^−1^), −NH_2_ wagging (≈1298 cm^−1^), and N−N stretching (≈1100 cm^−1^) models of N_2_H*_y_* species, respectively.

Furthermore, to evaluate the role of electron‐donating ligands in desorption of critical intermediates during conversion of N_2_ to NH_3_, subsequent NRR steps were investigated using various Te‐based catalysts following an optimal reduction path (Figure S9, Supporting Information). When NRR follows alternating path, the protonation reaction alternately occurs between two N atoms, resulting in the release of NH_3_ in the sixth step. In agreement with DRIFT spectrum result, negative Δ*G* values for *N2 protonation step using TeO_2_ or TeOSe catalysts implied that the activation of N_2_ can be proceeded spontaneously. However, potential‐limiting step (Δ*G*) of N_2_ fixation on Te/TeO_2_ in alternating mechanism is transformation of *NHNH group. At zero electrode potential (*U* = 0), an energy barrier of ≈2.42 eV was required for the protonation of *NHNH. Remarkably, SeO ligand with large charge buffering capacity at central sites is crucial to the catalytic N_2_‐to‐NH_3_ conversion process, thereby buffering the oxidation states of Te centers for better desorption of N‐species (Figure 3d). This barrier can be lowered after doping with a certain amount of Se, with an energy barrier decreased to ≈0.98 eV in *NHNH protonation step. However, after increasing Se concentration, the potential‐limiting steps (2.24 eV) for the excess Se‐incorporated Te/TeO_2_ were protonation of *N_2_ to *NNH, which mainly originated from weak chemical binding effect toward molecular N_2_ in the first step (Figure 3b‐III).

Proved by these theoretical illustrations, electrocatalytic NRR tests were then performed to evaluate catalytic activity of Se, O‐dual‐decorated Te nanorods.^25^ N_2_ was primarily scrubbed to remove background NH_3_ or NO*_x_* contaminants.^26^ As shown by linear sweep voltammetric curves in **Figure** **4**a, current densities from Te‐containing catalysts were initiated at 0 V under N_2_ saturation, which were increased in comparison to that in Ar. Although Te NWs exhibited much higher current density, net current density of STRs for the N_2_ reduction was much higher than that using Te catalyst under various potentials, which were ascribed to N_2_‐to‐NH_3_ conversion and side reactions inducing gas adsorption to catalyst and polarization, etc.^27^ NRR using the STRs as catalyst was further investigated with N_2_ bubbling in cathodic chamber, where H^+^ in electrolyte was reacted with N_2_ to form NH_3_. As shown in Figure 4b, in sharp contrast to efficiency value of ≈8.77% and ≈0.06% for Te NWs and TeO_2_ powder under a potential of −0.3 V versus reversible hydrogen electrode (RHE), FE of optimized STRs catalyst for NH_3_ production increased to ≈24.56% with a partial current density of −0.56 mA cm^−2^, almost achieving a new record using a noble‐metal‐free catalysts. Due to the side reactions, FE values gradually decreased at more negative potentials. At a potential of −0.3 V versus RHE, STRs exhibited an average yield of ≈21.54 µg h^−1^ mg^−1^ for NH_3_ production, which was ≈3.5 and ≈5.8 times as high as that using pure Te NWs and TeO_2_, respectively. Due to the weaker chemical bonding effect, NH_3_ production catalyzed by heavily Se, O‐coated Te catalysts was decreased with the growth of Se concentration (Figure S10, Supporting Information).

**Figure 4:**
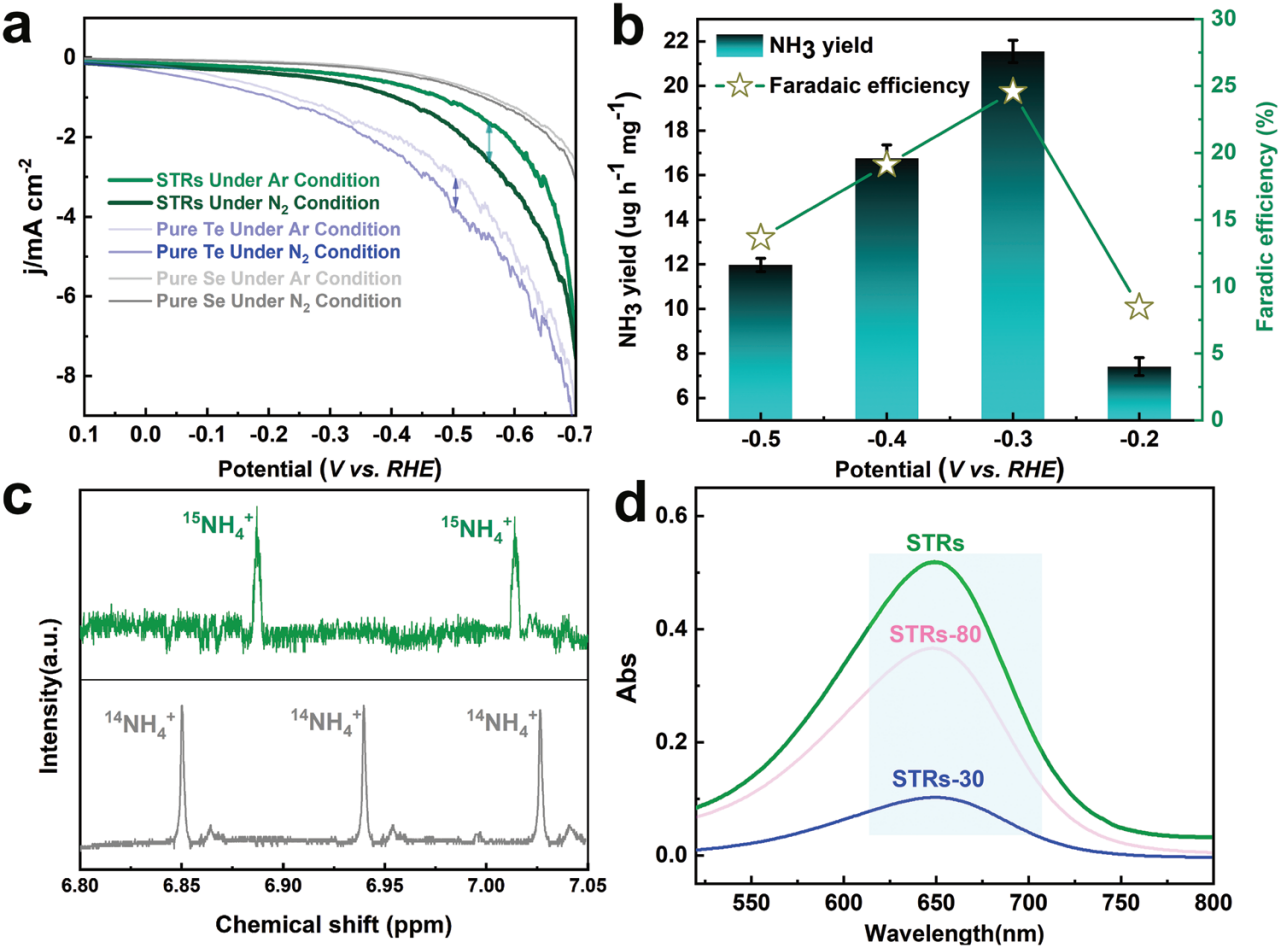
Electrochemical reduction of N_2_ to NH_3_. a) The linear sweep voltammetric curves at a scan rate of 2 mV s^−1^ for various catalysts in N_2_ (dark line) or Ar (pale line) saturated 0.1 m HCl aqueous solution. Note: The pertinent electrochemistry data in this work were repeated three times. b) Yield rate of NH_3_ production and FEs at each given potential for 2 h. c) NMR spectra of ^1^H for electrolytes after NRR test using ^15^N_2_ (top) and ^14^N_2_ (bottom) as feeding gas. d) UV‐vis absorption spectra of the electrolyte after electrolysis at −0.3 V using Te‐containing catalysts.

In order to confirm that NH_3_ production over Te‐based catalysts was generated through the electrochemical reduction process, control experiments were subsequently carried out. Isotopic labeling experiment using ^15^N_2_ enriched gas was performed to confirm that N source of NH_3_ was derived from supplied N_2_.^28^ As depicted by ^1^H NMR spectra, the two peaks corresponding to ^15^NH_4_^+^ exhibited a distinguishable chemical shift in contrast to ^14^N reference (Figure 4c), confirming that NH_3_ synthesized is resulted from the supply of N_2_. Due to the limited supply, velocity of ^15^N_2_ gas flow was ≈5 mL min^−1^ during labeling experiment, which was ≈60 times less than the experimental condition for reduction of ^14^N_2_. Thus, the relative low gas flow determined a low ^15^NH_4_^+^ concentration after reduction of ^15^N_2_, which resulted in low signal‐to‐noise ratio for ^15^NH_4_^+^. As shown by UV‐vis absorption spectra, the reaction in N_2_‐saturated electrolyte using carbon paper as electrode under −0.30 V for 2 h yielded NH_3_ production below detection limit. When the reactions using Te‐based catalysts proceeded in an Ar‐saturated electrolyte, UV‐vis spectra evidenced that no NH_3_ was detected (Figure S11, Supporting Information), which could eliminate interference of ammonia solution in the preparation of catalyst. Further, when reaction proceeded in an N_2_‐saturated electrolyte at −0.30 V versus RHE for 2 h, the UV‐vis absorption spectra of the electrolyte containing indophenol indicator demonstrated a characteristic peak at ≈650 nm (Figure 4d), which was another proof of NH_3_ formation via electroreduction of N_2_ in STRs platform. As a critical element for practical application, durability of catalyst was meanwhile evaluated (Figures S12 and S13, Supporting Information). No significant fluctuation occurred in current intensity, nor was obvious decline in NH_3_ yield observed after ten times recycles in a long‐term process.

In summary, we here report an in situ interfacial engineering strategy to incorporate electron‐donating ‐SeO ligands onto HER‐inactive Te nanorod. Detailed characterizations confirmed that Te‐Se‐O bonds were successfully constructed in shell of Te support. Based on combined results of predications and N_2_ adsorption analysis, it was theoretically and experimentally verified that exposure of LP electrons in TeO_2_ shell of STRs can maximize the orbital overlap with N_2_ for optimal N‐N bond activation. Meanwhile, the Gibbs free energy change indicated that Lewis‐base anchor ‐SeO ligands with strong electron‐donating character act as buffers for oxidation state variation of Te, thereby improving thermo‐dynamics of desorption of key intermediates in the N_2_ to NH_3_ conversion process. Under a low overpotential of −0.30 V versus RHE, a high FE of 24.56% and NH_3_ yield of ≈21.54 µg h^−1^ mg^−1^ were obtained in aqueous electrolyte under ambient conditions. Overall, in pursuit of NRR efficiency comparable to thermochemical NH_3_ synthesis processes, the work highlights the importance of featuring synergistic functionalities of bond activation and chemical states buffering in electrochemical N_2_ fixation, whereby achieving the efficient N‐N bond activation and optimal N‐intermediates adsorption–desorption. The findings also call for the reconsideration of alternative strategies to the realization of high effective catalysts in the domains of energy storage and conversions.

## Conflict of Interest

The authors declare no conflict of interest.

## Supporting information

Supplementary
