# Supplementary material for: Interfacial Engineering of SeO Ligands on Tellurium Featuring Synergistic Functionalities of Bond Activation and Chemical States Buffering toward Electrocatalytic Conversion of Nitrogen to Ammonia
Source: Adv Sci (Weinh). 2019 Aug 20;6(20):1901627. doi: 10.1002/advs.201901627 (PMC6794632; doi:10.1002/advs.201901627)
Supplement: Supplementary file 1 — Supplementary [file ADVS-6-1901627-s001.pdf]

Copyright WILEY-VCH Verlag GmbH & Co. KGaA, 69469 Weinheim, Germany, 2019.

ADVANCED SCIENCE
Open Access

Supporting Information

for *Adv. Sci.,* DOI: 10.1002/advs.201901627

Interfacial Engineering of SeO Ligands on Tellurium Featuring Synergistic Functionalities of Bond Activation and Chemical States Buffering toward Electrocatalytic Conversion of Nitrogen to Ammonia

Gong Zhang, Hang Xu, Yang Li, Chao Xiang, Qinghua Ji, Huijuan Liu, Jiuhui Qu, and Jinghong Li**

Supporting Information

Interfacial Engineering of SeO Ligands on Tellurium Featuring Synergistic Functionalities of Bond Activation and Chemical States Buffering toward Electrocatalytic Conversion of Nitrogen to Ammonia

Gong Zhang,[a,b] Hang Xu,[c] Yang Li,[b,c] Chao Xiang,[a,b] Qinghua Ji*,[a,b] Huijuan Liu,[a,b] Jiuhui Qu [a,b, e], Jinghong Li*,[d]

[a] School of Environment, State Key Joint Laboratory of Environment Simulation and Pollution Control, Tsinghua University, Beijing 100084, China

[b] Center for Water and Ecology, Tsinghua University, Beijing 100084, China

[c] Key Laboratory of Integrated Regulation and Resource Development on Shallow Lake of Ministry of Education, College of Environment, Hohai University, Nanjing 210098, China

[d] Department of Chemistry, Key Laboratory of Bioorganic Phosphorus Chemistry & Chemical Biology, Tsinghua University, Beijing 100084, China

[e] Key Laboratory of Drinking Water Science and Technology, Research Center for Eco-Environmental Sciences, Chinese Academy of Sciences, Beijing 100085, China

Detailed experimental sections

Preparation of the Te-containing materials. First, the ultrathin Te nanowires were prepared using previously developed method. Briefly, 150 mg Na_2TeO_3 and 1.0 g PVP and were dissolved in DIW under magnetic stirring to achieve a homogeneous solution at room temperature. 3.3 mL of aqueous ammonia solution were then added to adjust pH value for the disproportionation reaction. The mixture was transferred to a Teflon vessel held in a stainless steel vessel, which was closed and placed in an oven and heated at 160 °C for 3 h, and then the obtained sample after centrifugation was Te nanowires.

Se, O-decorated nanorods with different Te/Se molar ratios can simply be synthesized by tuning the molar ratios between Te and Se precursors. Typically, various amounts (30, 50, 80, and 100 mg) of Na_2SeO_3 were respectively dissolved into Na_2TeO_3 and PVP containing solutions under vigorous stirring. Similarly, 3.3 mL of aqueous ammonia solution was also added to change pH value, and 1.7 mL of hydrazine hydrate was dropwisely added into the as-obtained solutions. After reaction in a sealed Teflon vessel at 160 °C for 3 h, the depositions were centrifuged and denoted as STRs, STRs-30, STRs-80, and STRs-100, respectively.

Characterization. Morphology of the as-obtained Te-based samples were observed using a Zeiss Field Emission Scanning Electron Microscopy (FESEM). The phase compositions were determined by an X-ray diffractometer with Cu Kα radiation (λ = 0.15406 nm). Information on lattice spacings and fringes was obtained using a high-resolution JEM-ARM200F TEM/STEM. XPS experiments were performed with

PHI5000 Versa Probe system (Physical Electronics, MN), and binding energy was calibrated against reference of C1s peak at 284.8 eV. O and Se K-edge X-ray absorption fine structure spectroscopy (XAFS) were respectively carried out at 4B7B beamline at Beijing Synchrotron Radiation Facility (BSRF) China and Shanghai Synchrotron Radiation Facility (SSRF) China.

Nitrogen temperature programmed desorption (N_2-TPD) measurements were performed using a chemisorption apparatus. 20 mg of catalysts was placed in the glass tube, and were pretreated at 150 ^oC for 1 h, and cooled down to 50 ^oC. Adsorption of N_2 was conducted in a 99.999% N_2 gas flow for 3 h at 50 ^oC. After purging with He gas for 0.5 h, the sample was heated from 50 to 300 ^oC. TPD signal was recorded using a thermal conductivity detector. Diffuse reflectance Fourier transform infrared (DRIFT) spectra were recorded at room temperature in a diffuse reflectance cell with CaF_2 windows on a BRUKER tensor 27 FTIR spectrometer equipped with a vacuum.

Electrochemical measurements. Electrochemical measurements were carried out in three-electrode system at an electrochemical station (Gamry). 5 mg of catalyst and 40 μL of Nafion solution (5 wt%) were dispersed in 960 μL of water-ethanol solution with the volume ratio of 1:3 by sonication for 1 h. After that, 10 μL of dispersion was loaded onto a glassy carbon Electrode with the diameter of 5 mm (Note: N-free Nafion should not be a source of ammonia). For N_2 electrochemical reduction, the electrochemical measurements were carried out in an H-cell system which was separated by Nafion 115 membrane. The gases used in this work were primarily scrubbed (using water) to remove background NH_3 or NH_3 from exogenous sources,

as well as the NO_x contaminants. After that, the level of such species present was determined using the gas chromatography (Agilent 490-pro). As shown in following **Figure**, two obvious peaks at the retention time ~1.5 min and ~1.5 min were respectively ascribed to O_2 and N_2 in channel I, while only the peak assigned to mixture of O_2 and N_2 can be observed in channel II. This indicates that contaminants of NO, NO_2 and NH_3 can be excluded before electrocatalytic reduction step.

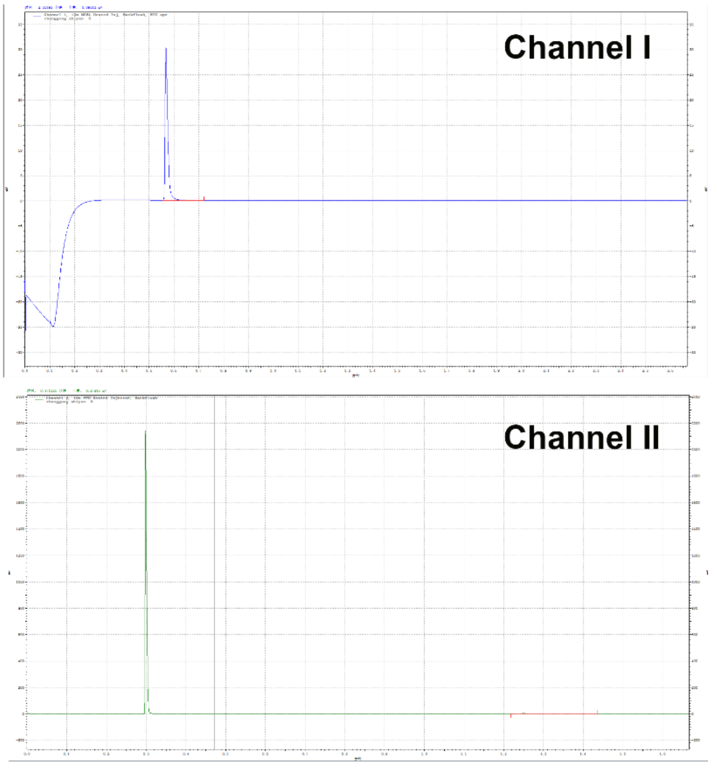

Ag/AgCl electrode and Graphite rod were applied as reference electrode and counter electrode, respectively. All potentials in this study were measured against Ag/AgCl reference electrode and converted to RHE reference by $E_{RHE} = E_{Ag/AgCl} + 0.21\ V + 0.0591 \times pH$. N_2 electrochemical reduction was conducted in N_2-saturated 0.1 M HCl solution. After N_2 was purged into HCl solution for at least 30 min to remove residual air, controlled potential electrolysis was performed at applied potentials for 2 h.

Determination of ammonia. The concentration of ammonia was determined by the indophenol blue approach. 2 mL of 1 M NaOH solution containing salicylic acid and sodium citrate was added into 2 mL of electrolyte after NRR, followed by addition of 1 mL of 0.05 M NaClO and 0.2 mL of $C_5FeN_6Na_2O$. The absorption spectrum was measured using an UV-vis spectro-photometer. The concentration of indophenol blue was determined using absorbance at wavelength of 655 nm. Concentration-absorbance curve was calibrated using standard ammonia chloride solution with a serious of concentrations. Fitting curve (y=0.065 x + 0.033, R^2=0.997) shows good linear relation of absorbance value with NH_3 concentration by three times independent calibrations.

Faradaic efficiency for NH_3 production was calculated at a given potential as follow:

$FE = C_{NH3} \times V \times N \times F/Q$

C_{NH3}: the measured NH_3 concentration;

V: volume of the electrolyte;

N: the number of electrons transferred for product formation, which is 3 for NH_3;

F: Faraday constant, 96485 C mol^{-1};

Q: quantity of electric charge integrated by i-t curve.

To identify the source of ammonia, the isotopic labeling experiment using $^{15}N_2$ gas (99% $^{15}N \equiv ^{15}N$, Shanghai Yuanneng Biotechnology Co., Ltd.) as the feeding gas was thereafter performed. In view of limited supply and high expense of $^{15}N_2$, the velocity of $^{15}N_2$ gas flow was set at 5 mL min^{-1}. After electrolytic reaction for 6h,

$^{15}NH_4^+$-contained electrolyte was detected by using ^1H (nuclear magnetic resonance, 600 MHz) NMR spectroscopy.

Calculation Details

The surfaces of Te (111) has been built, where the vacuum space along the z direction is set to be 15 Å, which is enough to avoid interaction between the two neighboring images. Then the Te_3O_6, Te_5Se_4, and $Te_3Se_3O_3$ clusters have been loading on the surface of Te(111). The bottom two atomic layers were fixed, the top three atomic layers and clusters were relaxed adequately. The first principles calculations in the framework of density functional theory were carried out based on the Cambridge Sequential Total Energy Package known as CASTEP. The exchange–correlation functional under the generalized gradient approximation (GGA) with norm-conserving pseudopotentials and Perdew–Burke–Ernzerhof functional was adopted to describe the electron–electron interaction. An energy cutoff of 750 eV was used and a k-point sampling set of 5 × 4 × 1 were tested to be converged. A force tolerance of 0.01 eV $Å^{-1}$, energy tolerance of 5.0×10^{-7}eV per atom and maximum displacement of 5.0×10^{-4} Å were considered.

According to the method presented by Nørskov, the Gibbs free energy diagrams were estimated by the following equation,

$$\Delta G_i = \Delta E_i + \Delta ZPE_i - T\Delta S_i - eU$$

where ΔE is the energy change between the reactant and product obtained from DFT calculations; ΔZPE is the change of zero point energy; T and ΔS denote temperature and change of entropy, respectively. i represents three intermediates; U is the potential

measured against normal hydrogen electrode (NHE) at standard conditions; *e* is the transferred charge, *T* is the temperature with unit K). In here, *T* = 300K was considered.

Supplementary Figures

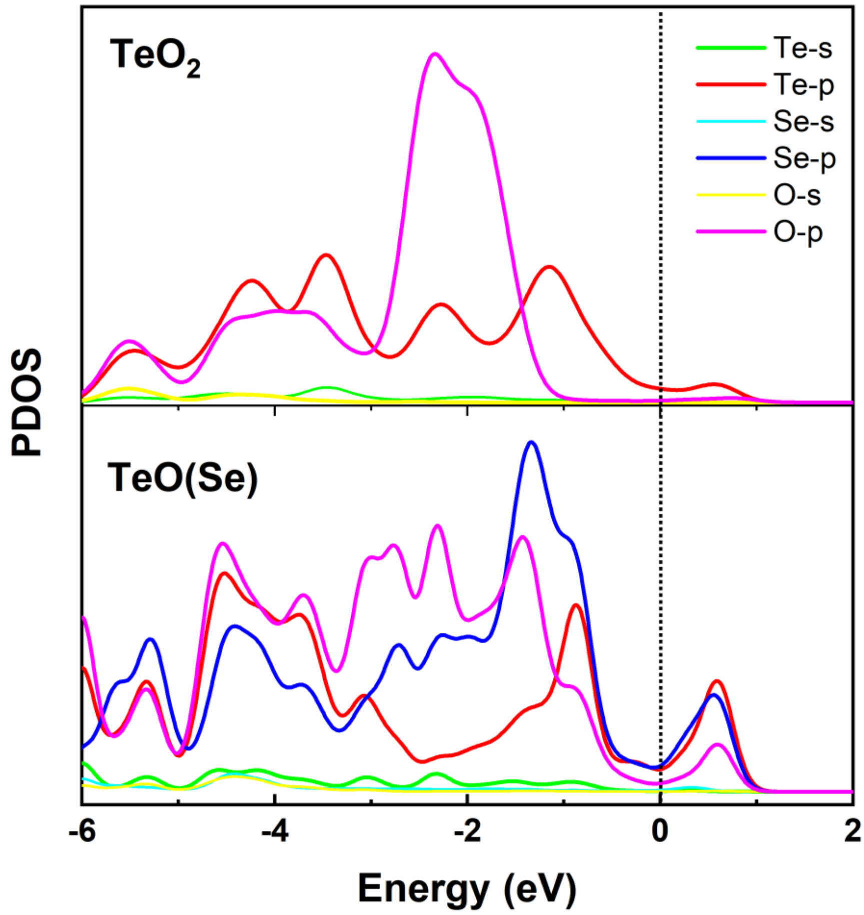

Figure S1. Calculated Partial density of states (PDOS) of the Te/TeO_2 and Te/TeO (Se). In the presence of Se, the hybridization between the Te 5p-orbital and O 2p-orbital is remarkably enhanced after introducing Se, leading to the increased density of states (DOS) nearby Fermi Level, which brings about more charge carriers, thereby determining the increased intrinsic electro-conductivity after introduction of Se.

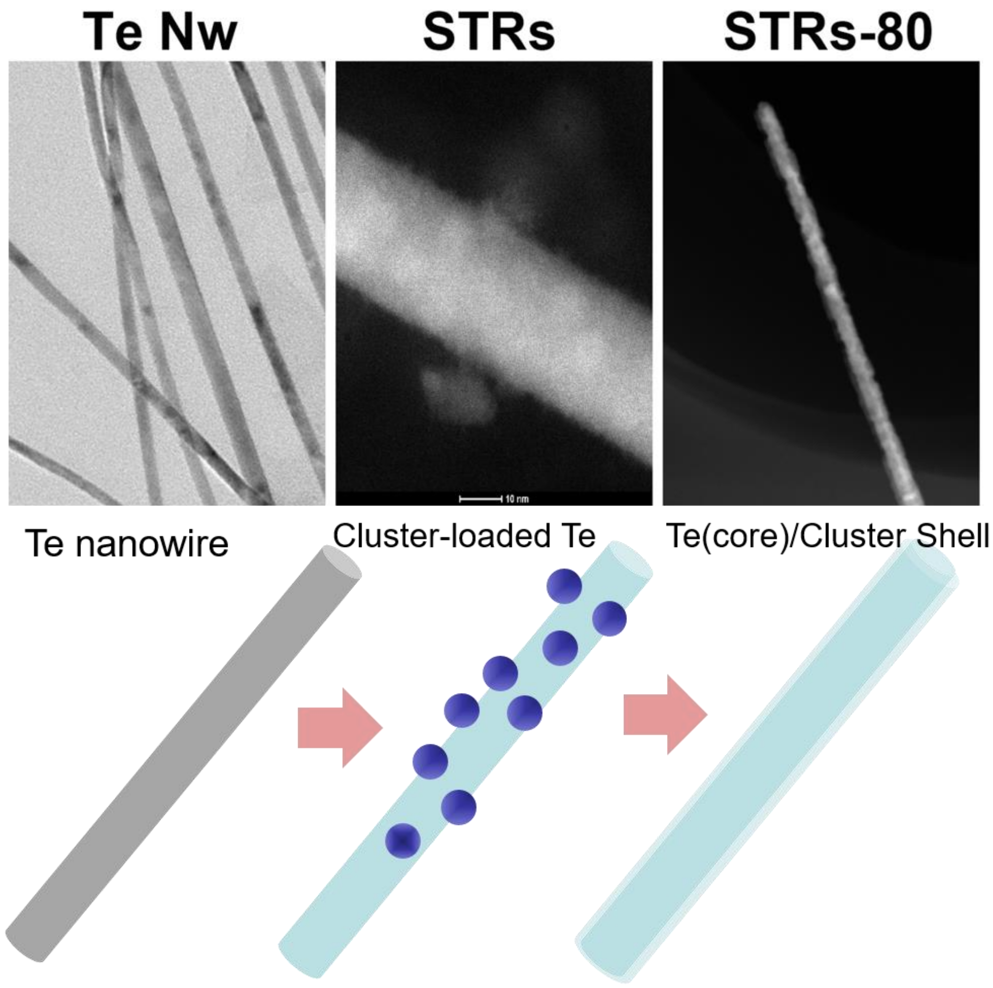

Figure S2. Illustration the formation mechanism of STRs. Considering fact that the radius of Se, O atoms are smaller than that of Te, O, Se atoms can be easily inserted into Te. On the basis of the phase diagram of O, Se, and Te are completely miscible in each other. When the aging temperature was increased to 160°C, O, Se atoms are initially released from precursors and inserted into the rigid Te nanowires. Under low concentration, the Se-O-Te clusters were loaded on Te skeleton, which was eventally converted into a shell structure after the addition of excess amount of Se precusors.

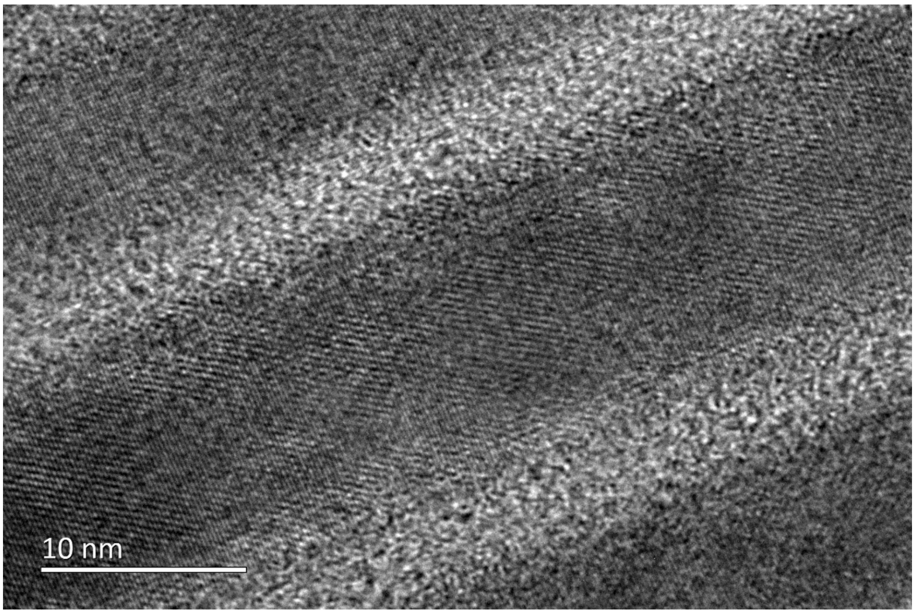

Figure S3. High-resolution TEM (HRTEM) of image of the pristine Te NWs. The image evidenced that the Te NWs have a smooth surface along length and the axis of the nanowire was along a [001] direction.

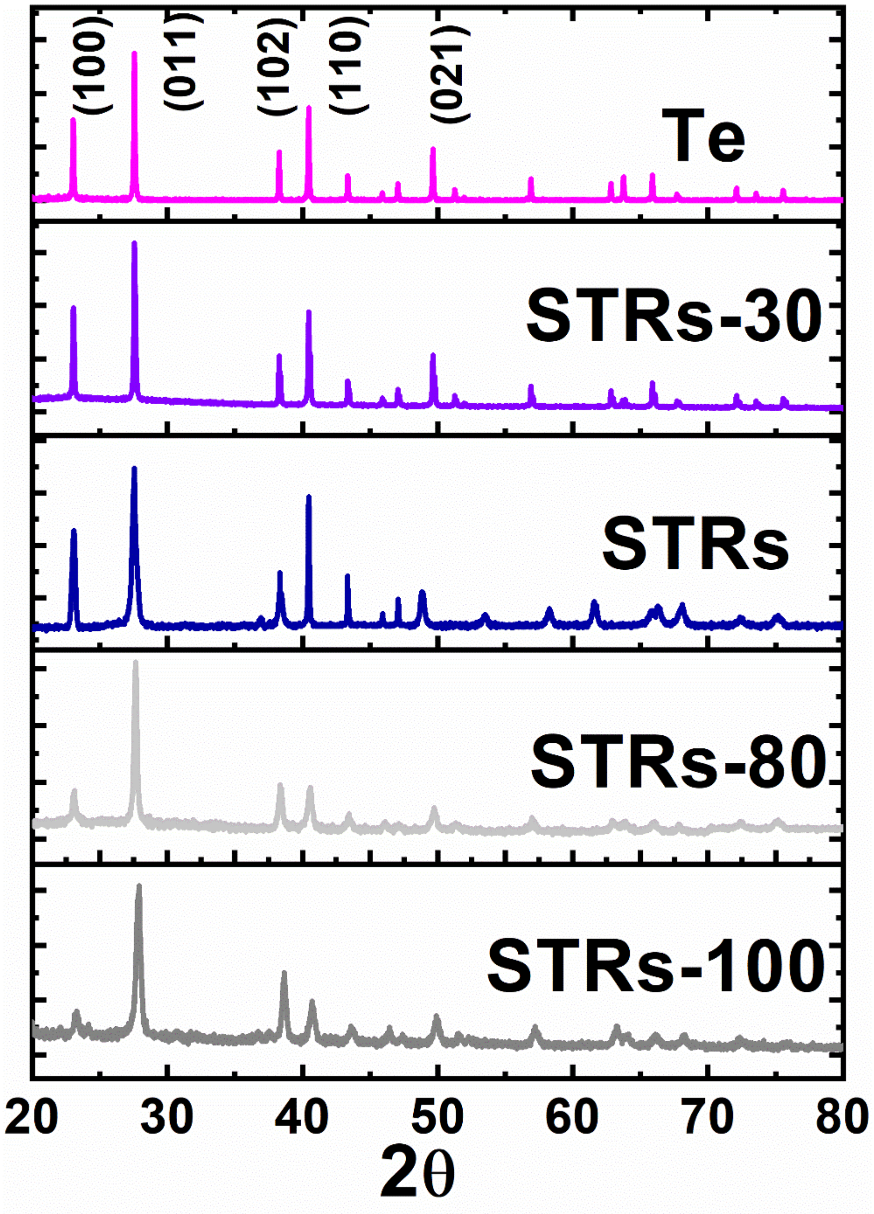

Figure S4. XRD patterns of Te-based materials a in the presence of different amount of Na_2SeO_3. The characteristic peaks of the XRD pattern for all samples are well indexed to the Te crystal, indicating that the *in-situ* incorporation of trace amount of Se, O on shell had no effect to its original crystal phase. However, with increasing of the amount of Na_2SeO_3, the decreased peak intensities were ascribed the exess Se, O incoporation.

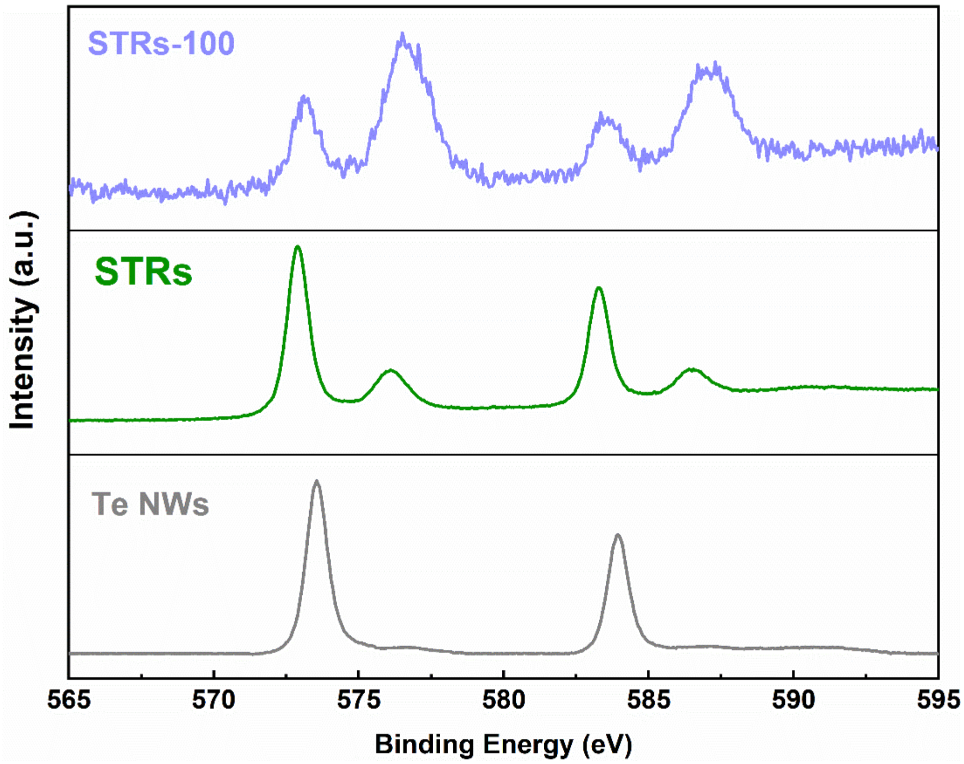

Figure S5. High-resolution XPS spectra of Te for pristine Te NWs and different STRs products, respectively. The intensities of peak ~575.95 eV assigned to Te^{4+} were remarkably increased with the growth of Se, suggesting that amount of surface Se or O decoration increased with the growth of the addtion of the Se precursor.

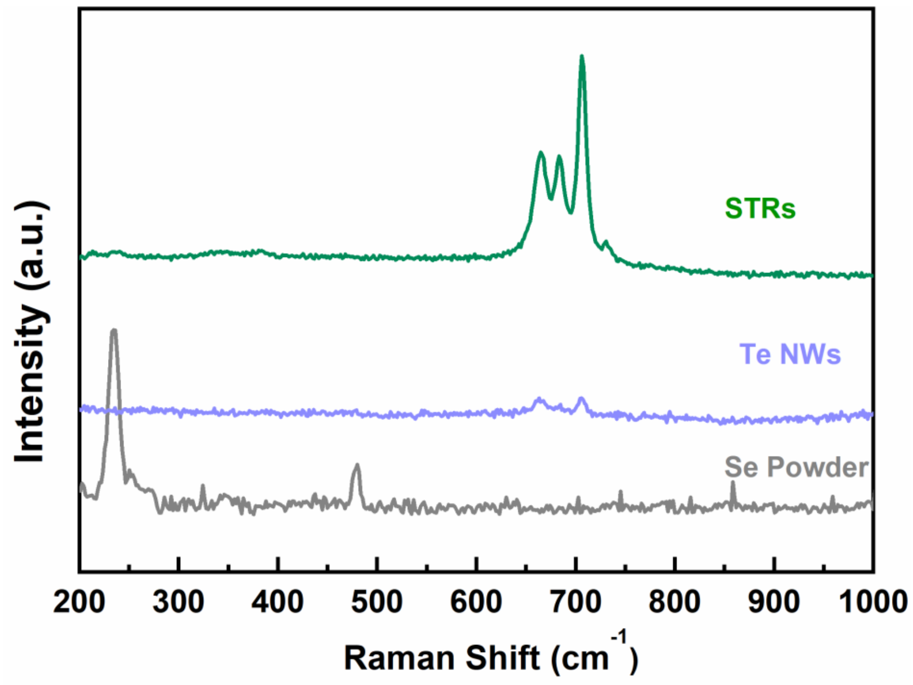

Figure S6. Raman spectra of the STRs, Te NWs, and Se powder, respectively. In contrast to Raman spectra for pristine Se, disappeared Raman characteristic peaks at 236 and 458 cm^{-1} excluded the presence of chain-structured and annular Se in STRs

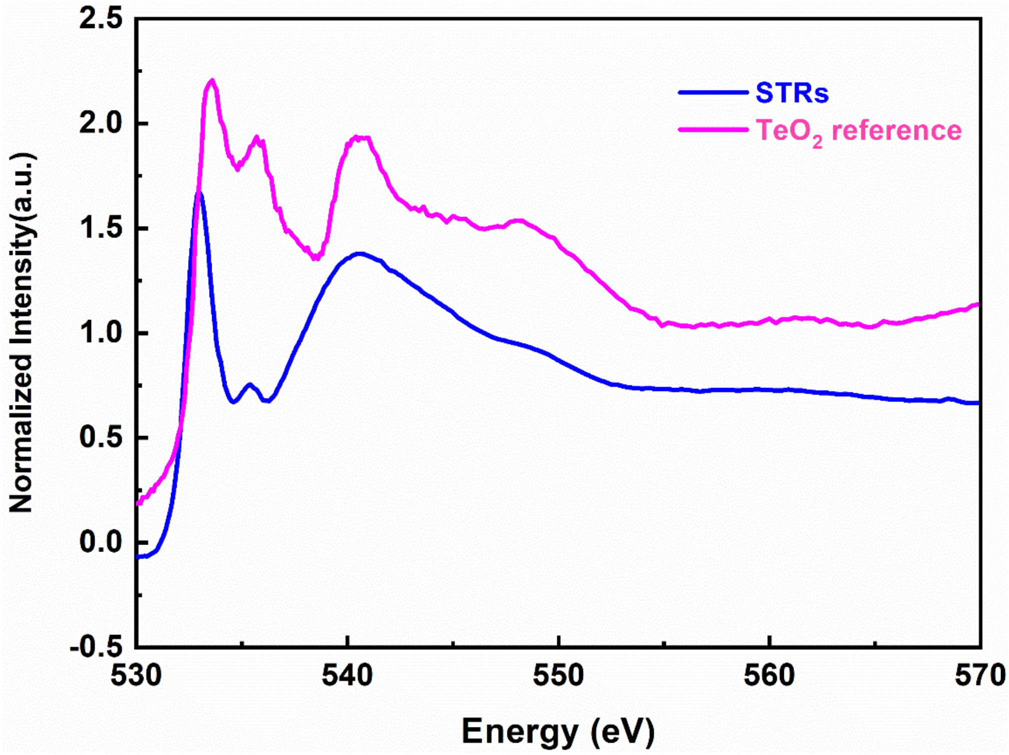

Figure S7. O K-edge XANES spectra of the Te-based materials. In general, the peaks at ~533, ~534–537, and ~545 eV were assigned to t_{2g}, e_g, and sp molecular orbits (MOs), respectively. In contrast to TeO_2 reference, the peak at ~533 eV assigned to the t_{2g} obviously decreased in STRs materials, which was due to charge redistribution induced by hybridization with Se atoms.

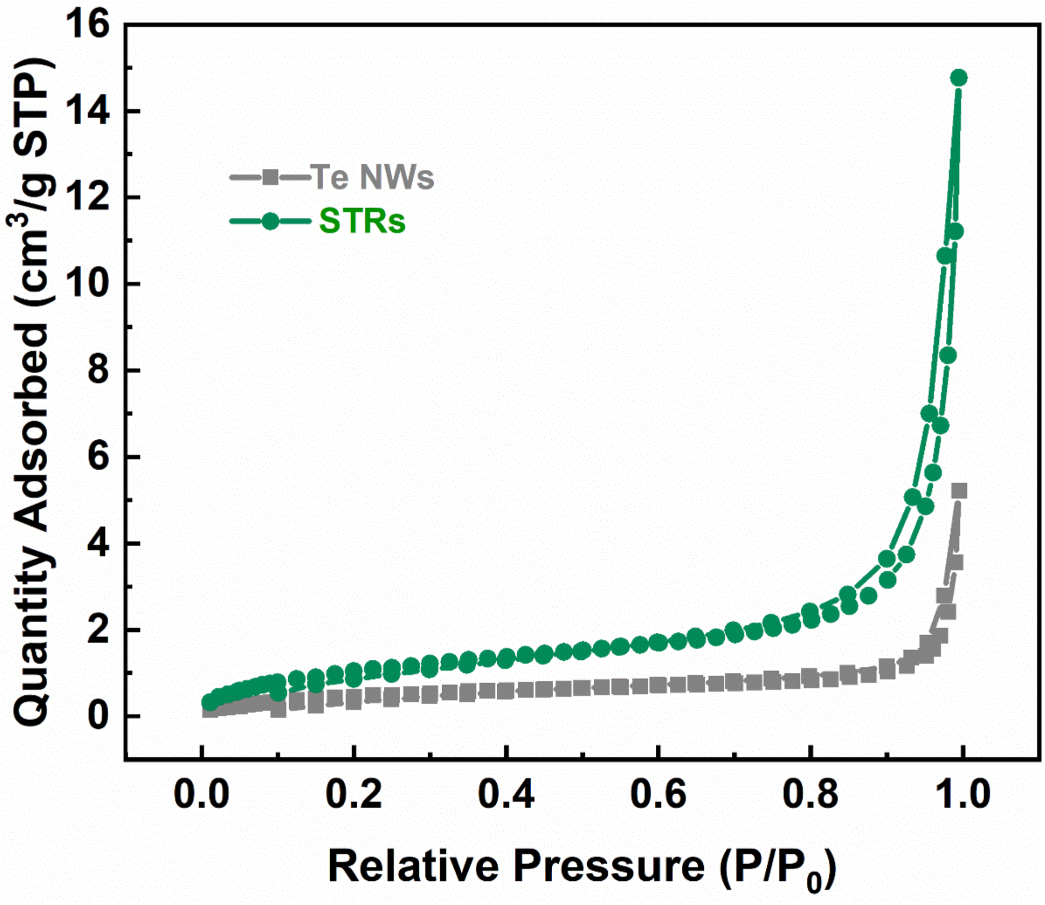

Figure S8. Nitrogen adsorption–desorption isotherms of the different catalysts. In contrast to the BET specific surface area of 1.5 m^2 g^{-1} for the pristine Te NWs, the hierarchical-structured STRs catalyst should have a positive influence on BET surface area, which was almost ~3 times higher than Te NWs.

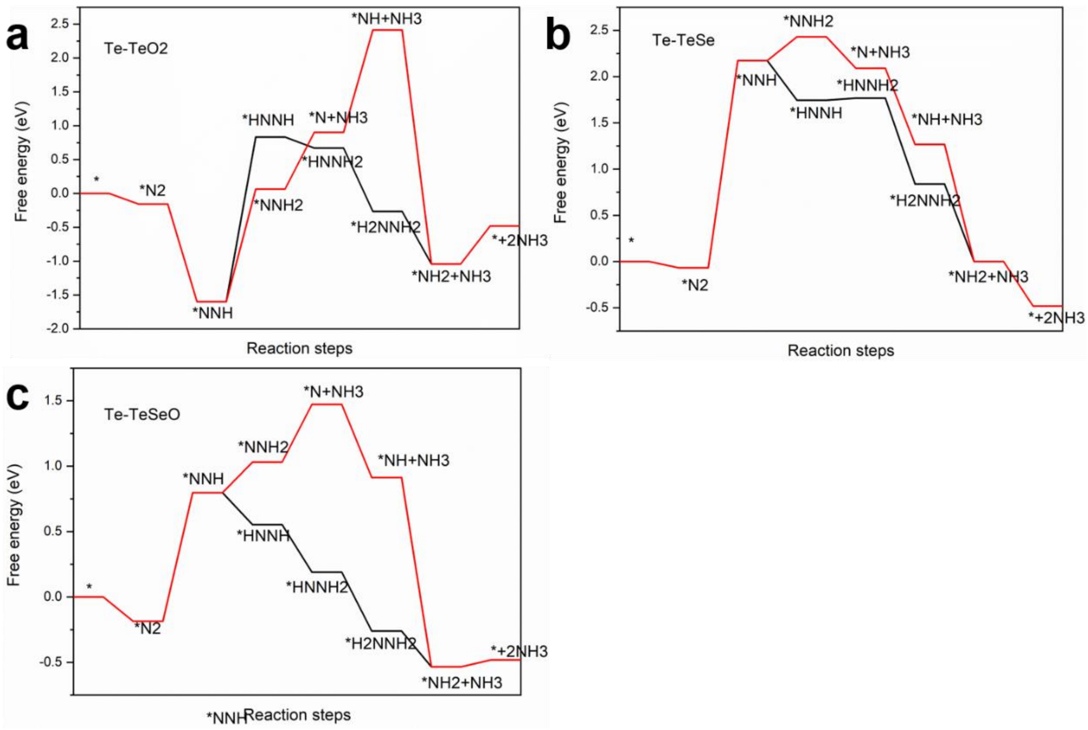

Figure S9. Illustration of free energy diagram for distal (red line) and alternavtive (red line) NRR pathways on Te-based samples. In distal pathway, the adsorbed N_2 will be hydrogenated by adsorbing a proton coupled with electron transfer, resulting in formation of N2H*. After that, (H^+ + e^-) consecutively reacts with distal N atom to formation of N2H*. NH_3 can be detached after interaction of prehydrogenated N site in N2H2*. After that, remaining N* species will be hydrogenated to second NH_3 via a similar path by another three protons coupled with electrons. In addition to TeO_2, in view of the smaller overpotential, NRR occurring on Te-based catalysts preferred to proceed through alternating mechanism.

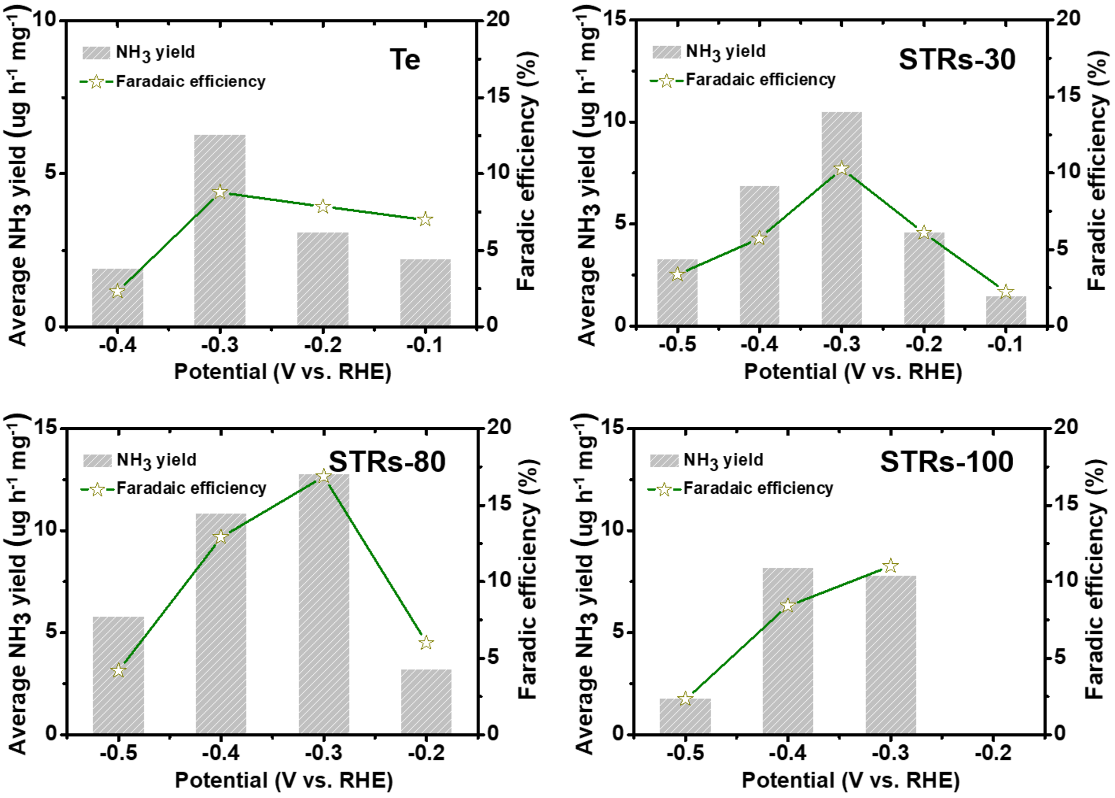

Figure S10. Catalytic performance of Te-contianing catalyts during electrocatalytic N_2 reduction process. Yield rate of NH_3 production and Faradaic efficiencies at each given potential for 2 h using different Te-based catalysts. Although pristine Te NWs exhibited the highest polarization cuurent, the relative low NH_3 production rate under various overpotential was mainly due to the sluggish kinetics of N_2 absorption and strong HER competion. In the presence of Se, NH_3 production rate increased to a peak value when suitiabe introduction of Se onto Te surface. However, due to chemical bonding effect, the NH_3 production catalyzed by the excessive Se-coated Te NWs catalysts were decreased.

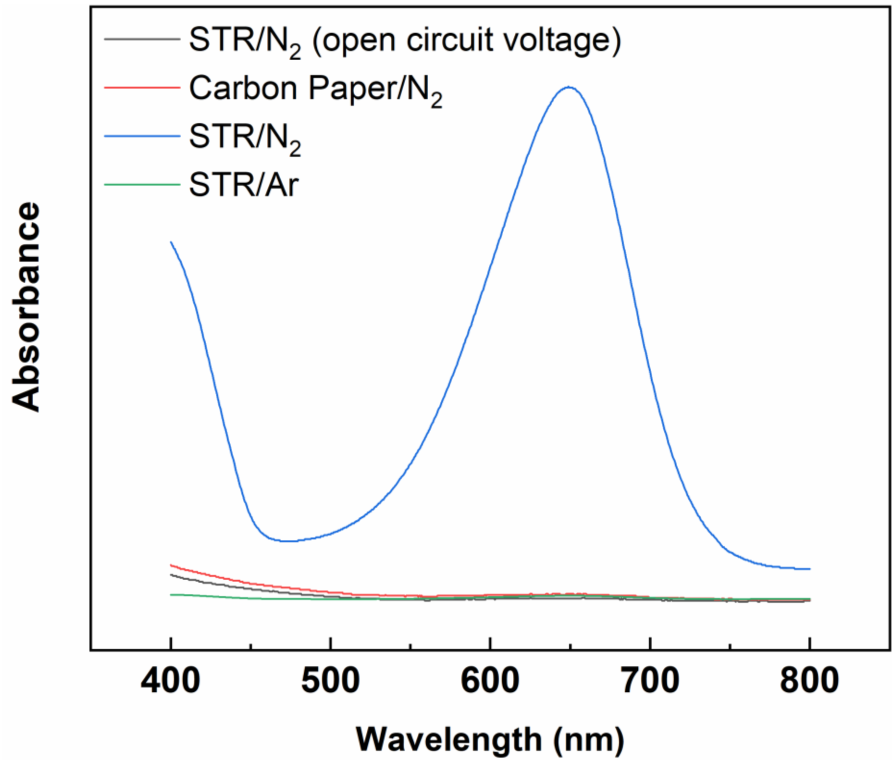

Figure S11. UV-vis absorption spectra of electrolyte after electrolysis at −0.3 V for 2h under control conditons. To verify that reduction product was generated via NRR catalyzed by Te-based catalysts, comparing experiments were performed using carbon paper as the working electrode. Ar/N_2 gas flow is introduced into an electrochemical reaction cell at potential of -0.3 V for 1 h. UV–vis absorption spectra presented no NH_3 product is detected in these conditions.

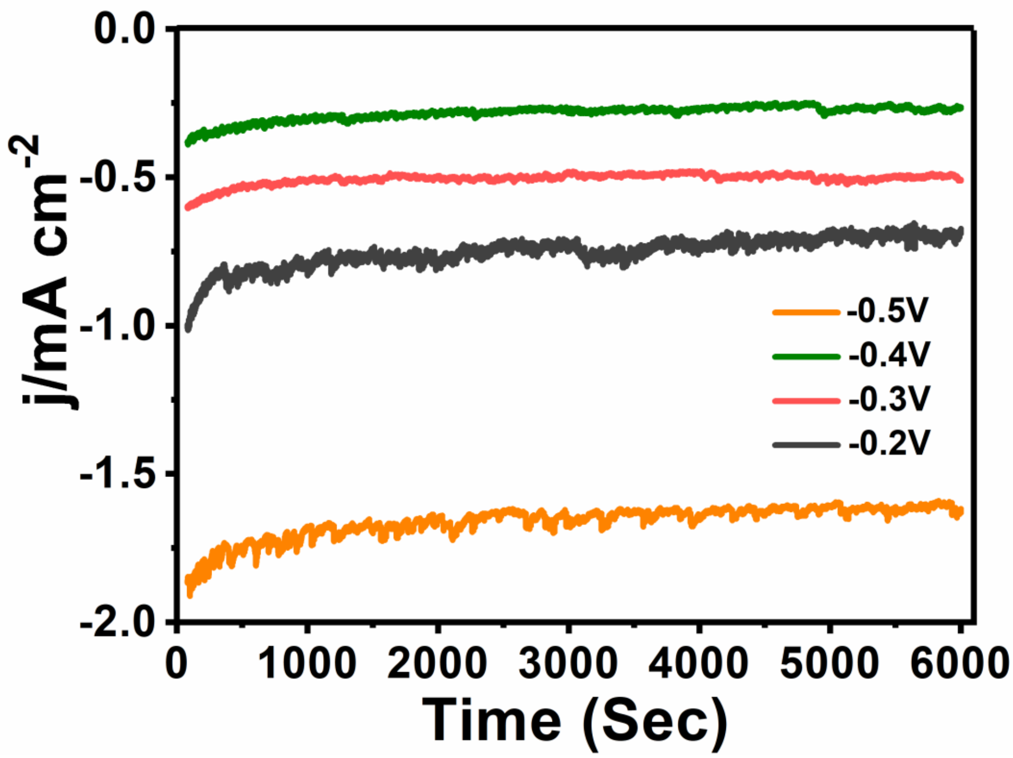

Figure S12. Durability test for the STRs toward N_2 electrochemical reduction at different given potentials.

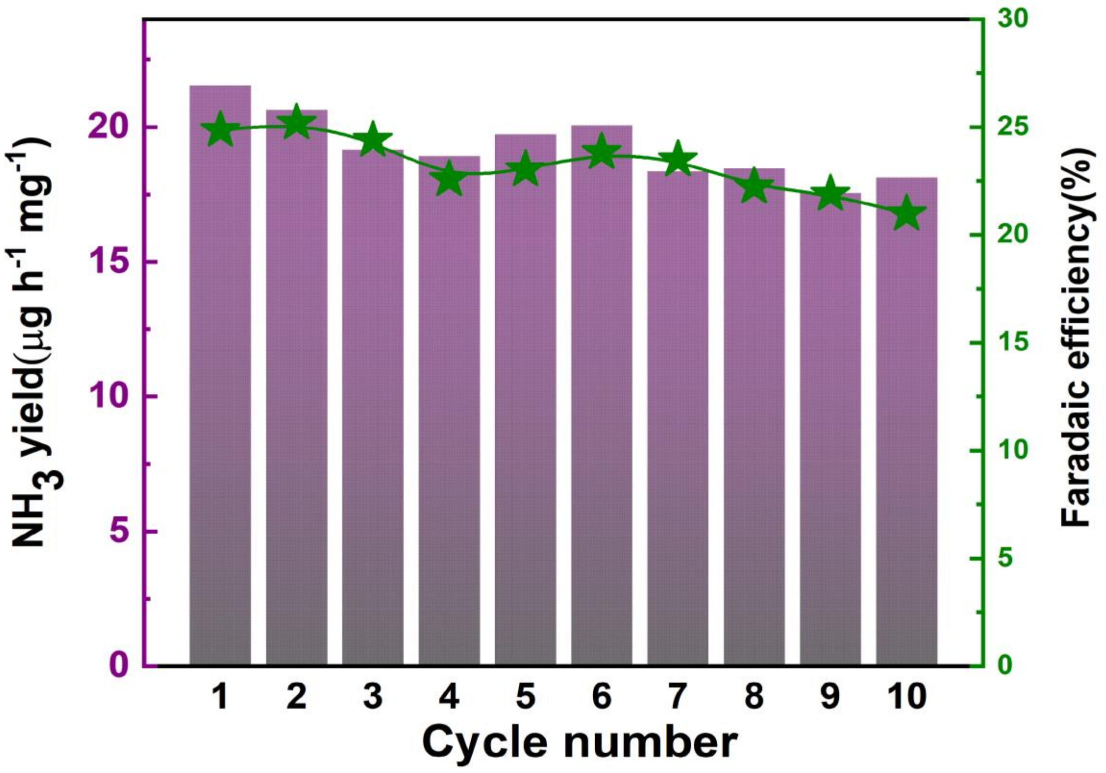

Figure 13. Cycling tests for the NRR using STRs catalyst.

Table S1. Idenficaiton of the mass fraction of Se in STRs using the inductively coupled plasma–mass spectrometry (ICP-MS) after the addtion of different amount of Se precursors

Number	Name	Amount of Se addtion	Mass fraction of Se
1	Te-NWs	0	ND
2	STRs-30	30 mg	~0.32%
3	STRs	50 mg	~0.53%
4	STRs-80	80 mg	~1.06%
5	STRs-100	100 mg	~1.18%
